# Supplementary material for: Adaptations to different habitats in sexual and asexual populations of parasitoid wasps: a meta-analysis
Source: PeerJ. 2017 Sep 12;5:e3699. doi: 10.7717/peerj.3699 (PMC5600175; doi:10.7717/peerj.3699)
Supplement: Supplemental Information 1 [file peerj-05-3699-s001.docx]

Figure 1

Studies included in quantitative synthesis (meta-analysis)
(n = 17 )

Studies included in qualitative synthesis
(n = 17 )

Full-text articles excluded, with reasons
(n = 6 )

Full-text articles assessed for eligibility
(n = 23)

Records excluded
(n = 0)

Records screened
(n = 23)

Records after duplicates removed
(n = 23)

Additional records identified through other sources
(n = 1 )

## Identification

## Eligibility

## Included

## Screening

Records identified through database searching
(n = 22 )
